# The effect of calcium dobesilate combined with hypoglycemic drugs in the treatment of cataract NPDR and its effect on fundus microcirculation and blood ICAM-1, MCP-1 and MIF levels

**DOI:** 10.5937/jomb0-37338

**Published:** 2023-10-27

**Authors:** XiaoMan Wang, Zhao Zhang, Yi Qing Ge, Xia Wu, Yin Chao Ma

**Affiliations:** 1 Dingzhou People's Hospital, Department of Pharmacy, Dingzhou City, China; 2 The Fourth Hospital of Zhangjiakou City (Ophthalmology Hospital), Department of Pharmacy, Zhangjiakou City, China; 3 Shijiazhuang Hospital of Traditional Chinese Medicine, Department of Pharmacy, Shijiazhuang City, China

**Keywords:** calcium dobesilate, combination therapy, cataract with nonproliferative diabetic retinopathy, fundus microcirculation, intercellular adhesion molecule 1, monocyte chemotactic protein 1, macrophage migration inhibitory factor, kalcijum dobesilat, kombinovana terapija, katarakta sa nonproliferativnom dijabetičnom nefropatijom, mikrocirkulacija fundusa, intracelularni adhezioni molekul 1, monocitni hemotaktični protein 1, makrofagni migracioni inhibitorni faktor

## Abstract

**Background:**

To explore the effect of calcium dobesilate combined with hypoglycemic drugs in the treatment of cataract complicated with non-proliferative diabetic retinopathy (NPDR) and its effects on fundus microcirculation, intercellular adhesion molecule 1 (ICAM-1), mono - cyte chemoattractant protein 1 (MCP-1), and macrophage migration inhibitory factor (MIF).

**Methods:**

From March 2019 to January 2021, a total of 114 patients with cataract and NPDR were included, and the patients were assigned into the control and the observation groups by random number table method, with 57 cases/group. The control was given hypoglycemic drugs, and the observation was given calcium dobesilate combined therapy. The therapeutic efficacy, blood glucose and blood lipid levels, fluorescein fundus angiography results, fundus microcirculation indexes, retinal neovascularizationrelated factors, and ICAM-1, MCP-1, and MIF levels before and after treatment were compared between the two groups.

**Results:**

The total effective rate of treatment in the observation was higher vs. the control (P < 0.05); Fasting blood glucose (FBG), 2 h postprandial blood glucose (2hPG), glycosylated hemoglobin (HbA1c), triglyceride (TG), total cholesterol (TC) and low density lipoprotein (LDL) in the observation after treatment were reduced vs. the control (P < 0.05); The number of micro-hemangiomas in the observation after treatment was less vs. the control, and the area of hemorrhage, the area of exudation and the thickness of the yellow plate were smaller vs. the control (P < 0.05); The resistance index (RI) value of the observation after treatment was lower than the control, and the end-diastolic blood flow velocity (EDV) and the peak systolic blood flow velocity (PSV) of the observation were higher vs. the control (P < 0.05). ICAM-1, MCP-1, MIF, vascular endothelial growth factor (VEGF) and insulin-like growth factor-1 (IGF1) in the observation after treatment were reduced vs. the control, but pigment epithelium-derived factor (PEDF) were higher vs. the control (P < 0.05); one case of gastrointestinal reaction took place in the observation, but no adverse reaction occurred in the control, and no clear difference exhibited in the incidence of adverse reactions between the two groups (P > 0.05).

**Conclusions:**

Calcium dobesilate combined with hypoglycemic drugs has good clinical efficacy in the treatment of cataract complicated with NPDR, which can effectively reduce the level of blood glucose and blood lipids, reduce inflammation, and mitigate the microcirculation of branch retinal vein occlusion lesions.

## Abstract (sr)

**Uvod:**

Cilj je bio da se istraži efekat kalcijum dobesilata u kombinaciji sa hipoglikemijskim lekovima u lečenju katarakte komplikovane neproliferativnom dijabetičkom retinopatijom (NPDR) i njenih efekata na mikrocirkulaciju fundusa, molekula intercelularne adhezije 1 (ICAM-1), monocitnog hemoatraktantnog proteina 1 (MCP-1) i faktor inhibitora migracije makrofaga (MIF).

**Metode:**

Od marta 2019. do januara 2021. godine uključeno je ukupno 114 pacijenata sa kataraktom i NPDR, a pacijenti su raspoređeni u kontrolnu i opservacionu grupu metodom tabele slučajnih brojeva, sa 57 slučajeva/grupi. Kontroli su davani hipoglikemijski lekovi, a posmatranoj kom binovana terapija kalcijum dobezilatom. Terapijska efikasnost, nivo glukoze u krvi i lipida u krvi, rezultati fluoresceinske angiografije fundusa, indeksi mikrocirkulacije fundusa, faktori povezani sa neovaskularizacijom retine i nivoi ICAM-1, MCP-1 i MIF pre i posle tretmana su upoređeni između dve grupe.

**Rezultati:**

Ukupna efektivna stopa tretmana u posmatranju bila je viša u odnosu na kontrolu (P < 0,05); Glukoza u krvi natašte (FBG), 2 h postprandijalne glukoze u krvi (2hPG), glikozilovani hemoglobin (HbA1c), trigliceridi (TG), ukupni holesterol (TC) i lipoprotein niske gustine (LDL) u opservaciji nakon tretmana bili su smanjeni u odnosu na kontrolu (P < 0,05); Broj mikrohemangioma u opservaciji nakon tretmana bio je manji u odnosu na kontrolu, a površina hemoragije, površina eksudacije i debljina žute ploče bili su manji u odnosu na kontrolu (P < 0,05); Vrednost indeksa rezistencije (RI) opservacije nakon tretmana bila je niža od kontrolne, a krajnja dijastolna brzina protoka krvi (EDV) i vršna brzina sistolnog krvotoka (PSV) posmatranja su bila veća u odnosu na kontrolnu (P < 0,05). ICAM-1, MCP-1, MIF, faktor rasta vaskularnog endotela (VEGF) i faktor rasta sličan insulinu-1 (IGF-1) u opservaciji nakon tretmana bili su smanjeni u odnosu na kontrolni, ali faktor koji potiče od pigmentnog epitela (PEDF ) bio je veći u odnosu na kontrolu (P < 0,05); jedan slučaj gastrointestinalne reakcije se desio u posmatranju, ali nijedna neželjena reakcija se nije javila u kontroli, i nije bilo jasne razlike u incidenci neželjenih reakcija između dve grupe (P > 0,05).

**Zaključak:**

Kalcijum dobesilat u kombinaciji sa hipoglikemijskim lekovima ima dobru kliničku efikasnost u lečenju katarakte komplikovane NPDR, što može efikasno da smanji nivo glukoze u krvi i lipida u krvi, smanji zapaljenje i ublaži mikrocirkulaciju okluzivnih lezija vena retine.

## Introduction

Non-proliferative diabetic retinopathy (NPDR) is a familiar chronic ocular microcirculation complication in diabetic patients [1]. Relevant studies have pointed out adhesion factors and chemokines are involved in the presence of NPDR at the molecular level [2]. Intercellular adhesion molecule-1 (ICAM-1) affiliates from the immunoglobulin superfamily, is mainly expressed in vascular endothelial cells, and takes on a momentous role in the adhesion of monocytes, lymphocytes and endothelial cells. Relevant studies have pointed out the elevation of ICAM-1 is closely linked with the process of insulin resistance [3]. Monocyte chemotactic protein 1 (MCP-1) is a member of the chemokine family, which can chemotactic monocytes and regulate vascular end o thelialrelated adhesion molecules. Macrophage migration inhibitory factor (MIF) is a pro-inflammatory factor secreted by T cells. Relevant studies have pointed out it and MCP-1 are involved in the presence and development of diabetic retinopathy [4]. At present, surgery is often applied to treat cataract, but clinical data clarify patients with NPDR will aggravate the degree of retinopathy after cataract surgery [5], so it is necessary to give effective treatment to patients with retinopathy at an early stage. Calcium dobesilate is a commonly applied antioxidant and microcirculation protector, which can improve capillary permeability and reduce platelet aggregation, thereby declining blood viscosity. However, its effect on CAM-1, MCP-1, and MIF in patients is still unknown nowadays. Therefore, the purpose of this study was to figure out the effect of calcium dobesilate combined with hypoglycemic drugs in the treatment of cataract complicated with NPDR and the effect on fundus microcirculation, CAM-1, MCP-1 and MIF, providing reference for the clinical treatment of this disease.

## Materials and methods

### Clinical data

From March 2019 to January 2021, a total of 114 patients with cataract and NPDR were enrolled, and the patients were assigned into the control and the observation groups by random number table method, with 57 cases/group. No clear difference exhibited in the above data between the two groups (*P* > 0.05 Table 1).

**Table 1:** Comparison of general data between the two groups.

Classification	Observation group (n = 57)	Control group (n = 57)	*χ^2/t^*	P
Age (years)	63.01±5.39	64.21±5.78	0.552	0.583
Gender (cases)			0.583	0.445
Male	32	36		
Female	25	21		
Duration of diabetes (years)	8.64±1.13	8.91±1.06	0.600	0.551
Fundus lesions staging (cases)			0.684	0.710
Stage I	21	25		
Stage II	16	13		
Stage		20	19	
Cataract Staging (cases)			0.371	0.542
Initial stage		19	16	
Immature stage	38	41		
Combined underlying diseases				
Hyperlipidemia	13	14	0.049	0.826
Hypertension	9	6	0.691	0.406
Family history of diabetes (cases)	6	4	0.438	0.508

### Inclusion criteria

Complying with the diagnostic guidelines for cataracts in Guidelines for the Prevention and Treatment of Cataracts [6]; In line with the diagnostic criteria for NPDR in Guidelines for Clinical Diagnosis and Treatment of Diabetic Retinopathy in China (2014) [7]; Patients with complete clinical data.

### Exclusion criteria

Patients with severe heart, liver and kidney dysfunction; proliferative diabetic retinopathy; combined severe heart diseases; developing infectious diseases within 3 months before enrollment; combined severe essential hypertension; poor medication compliance; combined immune system diseases; patients allergic to the drugs applied in this study.

### Methods

The control group was given conventional hypoglycemic drugs, acarbose (Bayer Health Care Co., Ltd., batch number: H19990205, specification: 50 mg*30 tablets, dose: 50 mg/time, 3 times/d) + metfor min tablets (Sino-American Shanghai Squibb Phar maceutical Co. LTD, batch number: National Me dicine Zhunzi H20023370, specification: 0.5g*20 tablets, dose: 0.5 g/time, 2 times/d), with a total of 6 months of treatment. The observation group was given calcium dobesilate (Hainan Linheng Pharmaceutical Co., Ltd., batch number: H20080644, specification: 0.5g*36 tablets) combined therapy, with the dose of 0.5 g/time, 3 times/d, a total of 6 months' treatment.

### Observation indicators

(1) Clinical efficacy [8]: markedly effective: blood sugar returned to normal and visual acuity improved by more than 2 lines, and clinical symptoms disappeared; Effective: blood sugar was clearly improved and visual acuity improved by more than 1 line, symptoms were apparently relieved; Ineffective: visual acuity improved < 1 line, and symptoms were not be improved. (2) Blood glucose and blood lipid levels: Glucose oxidase was employed to measure fasting blood glucose (FBG) and 2-hour postprandial blood glucose (2hPG) of patients before and 6 months after treatment. Glycosylated hemoglobin (Hemoglobin A1c, HbA1c) in patients before treatment and after 6 months of treatment were tested by liquid-phase method; Roche Modular DPP automatic biochemical analyzer was applied, and ultraviolet enzyme method was employed to detect Triglyceride (TG) in patients before treatment and after 6 months of treatment. Total cholesterol (TC) and low-density lipoprotein (LDL) were tested by cholesterol oxidase. (3) Fluorescein fundus angiography results: Before treatment and after 6 months of treatment, the patients were subjected to fluorescein fundus angiography with a German FFA/ICG fluorescence contrast instrument, and the contrast agent was fluorescein sodium (Guangzhou Star Pharmaceutical Co., Ltd., batch No.: Chinese medicine Zhunzi H44023401). (4) Fundus microcirculation indicators: The U.S. GELOGIQ p3 color Doppler ultrasound diagnostic instrument was employed to detect end diastolic velocity (EDV), peak systolic velocity (PSV), Resistance Index (RI) of the central retinal artery in the patient's eye before treatment and 6 months after treatment. (5). Retinal neovascularization-related factors: Rebo MK3 microplate reader and Enzyme-linked immunosorbent assay were applied to detect vascular endothelial growth factor (VEGF), Insulin-like growth factor 1 (IGF-1), pigment epithelium derived factor (PEDF) in patients before treatment and 6 months after treatment; The kit was provided by Nanjing Jinyibai Biotechnology Co., Ltd. (6) ICAM-1, MCP-1 and MIF: ELISA was employed to detect the serum MCP-1 and MIF in patients before treatment and after 6 months of treatment. The kits were provided by Bio-Rad Company of the United States; the serum ICAM-1 of the patients was detected by an OLYPUS AU2700 automatic biochemical analyzer. (7) Adverse reactions: The presence of gastrointestinal reactions and other adverse reactions in the two groups of patients during treatment was counted.

### Statistical processing

SPSS 22.0 software was employed to process data; Enumeration data were clarified as %, and differences between groups were compared by χ^2^ test; Measurement data were illustrated as (x̄±s) after normality test, and differences between groups were compared by t test. *P* < 0.05 emphasized obvious statistical meaning.

## Results

### Comparison of clinical efficacy between the two groups

The total effective rate of treatment in the observation was higher vs. the control (*P* < 0.05, Table 2).

**Table 2:** Comparison of clinical efficacy between the two groups (cases, %).

Groups	n	Clearly Effective	Effective	Ineffective	Total Effective Rate
Observation group	57	19	35	3	94.74 (54)
Control group	57	15	32	10	82.46 (47)
χ^2^					4.254
P					0.039

### Comparison of blood glucose and lipid levels between the two groups

**Figure 1:**
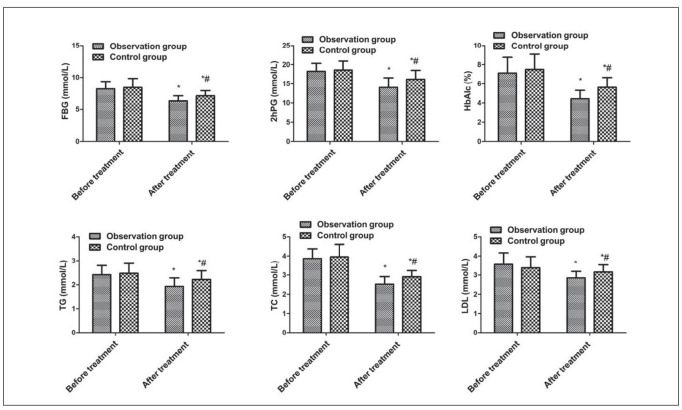
Comparison of blood glucose and lipid levels between the two groups. Vs. before treatment, *P < 0.05; vs. the observation after treatment, #P < 0.05.

No clear difference exhibited in FBG, 2hPG, HbA1C, TG, TC, and LDL of the two before treatment (*P* > 0.05). But these indicators were reduced after treatment of the two vs. before treatment, and the observation was lower vs. the control (*P* < 0.05, Figure 1).

### Comparison of fluorescein angiography results between the two

**Figure 2:**
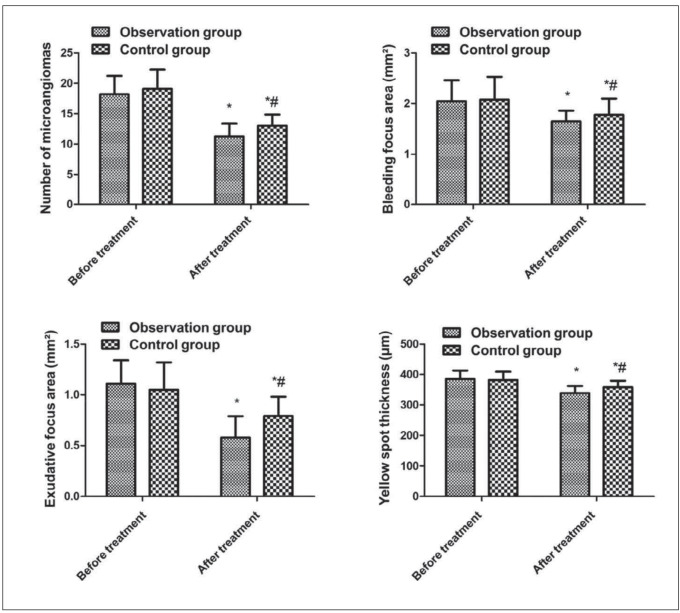
Comparison of fluorescein angiography results between the two. Vs. before treatment, *P < 0.05; vs. the observation after treatment, #P < 0.05.

Before treatment, no distinct differences were presented in the number of microvascular tumors, bleeding foci area, exudative foci area and yellow plate thickness between the two (*P* > 0.05). (P<0.05); After treatment, the number of microhemangiomas, the area of bleeding foci, exudation foci and thickness of yellow plate in the two were reduced and smaller vs. those before treatment, and the observation was smaller vs. the control (*P* < 0.05, Figure 2).

### Comparison of fundus microcirculation indexes between the two

**Figure 3:**
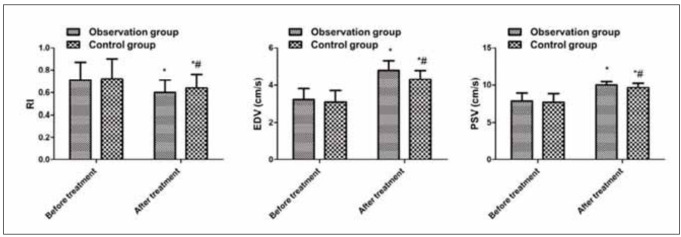
Comparison of fundus microcirculation indexes between the two. Vs. before treatment, *P < 0.05; vs. the observation after treatment, *#P* < 0.05.

No clear difference exhibited in RI, EDV and PSV between the two before treatment (*P* > 0.05); RI values in the two after treatment were declined vs. those before treatment, and the observation was smaller vs. the control (*P* < 0.05); EDV and PSV in the two after treatment were greater than those before treatment, and the observation was greater vs. the control (*P* < 0.05, Figure 3).

### Comparison of ICAM-1, MCP-1 and MIF between the two

**Figure 4:**
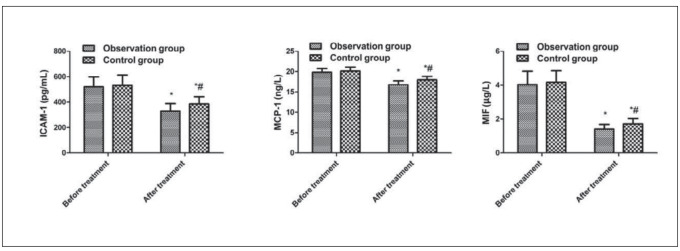
Comparison of ICAM-1, MCP-1 and MIF levels between the two. Vs. before treatment, *P < 0.05; vs. the observation after treatment, *#P* < 0.05.

No clear difference exhibited in ICAM-1, MCP-1 and MIF of the two before treatment (*P* > 0.05). But these indicators were reduced after treatment of the two vs. before treatment, and the observation was lower vs. the control (*P* < 0.05, Figure 4).

### Comparison of retinal neovascularization-related factors between the two

**Figure 5:**
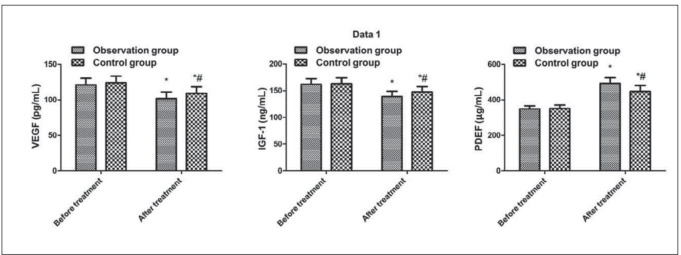
Comparison of retinal neovascularization-related factors between the two. Vs. before treatment, *P < 0.05; vs. the observation after treatment, *#P* < 0.05.

No clear difference exhibited in VEGF, IGF-1 and PEDF between the two before treatment (*P* > 0.05); VEGF and IGF-1 in the two after treatment were declined vs. those before treatment, and the observation was reduced vs. the control (*P* < 0.05); PEDF in the two after treatment was elevated vs. that before treatment, and the observation was prior to the control (*P* < 0.05, Figure 5).

### Comparison of the incidence of adverse drug reactions between the two

One case of gastrointestinal reaction took place in the observation, and no adverse reaction exhibited in the control. No clear difference exhibited in the incidence of adverse reactions between the two (*P* > 0.05).

## Discussion

Cataract combined with NPDR is caused by metabolic disorder clarified as the elevation of blood glucose in the patient's body, which enhances the glucose content of the lens, and the excess glucose can be converted into sorbitol, which saturates and accumulates in the lens, resulting in retinal swelling, turbidity and other symptoms. Patients with NPDR are prone to aggravation of retinopathy after cataract surgery, so early treatment of patients is vital. Calcium dobesilate is a commonly applied antioxidant and microcirculation protector, which can motivate the synthesis of substrate collagen, maintain the stability of retinal barrier function, enhance the flexibility of red blood cells, restrain thrombosis, mitigate retinal blood circulation, and reduce oxidation stress, retinal leukocytes and vascular endothelial adhesion, thereby protecting blood vessels [9] [10]. This study found the total effective rate of treatment in the observation was higher vs. the control, and the area of hemorrhage, the area of exudation and the thickness of the yellow plate after treatment were smaller vs. the control, indicating that the combined treatment can reduce the severity of retinopathy, mainly because hydroxy-benzene calcium sulfonate can reduce vascular proliferation and vascular permeability, thereby improving retinopathy.

In patients with NPDR complicated by cataract, the selective loss of retinal pericytes results in capillary hemangioma, which thickens the capillary basement membrane, results in the occurrence of fundus microcirculation disorders, and can cause blindness in severe cases [11] [12]. Relevant studies have pointed out calcium dobesilate treatment for patients with NPDR complicated with cataract can mitigate the microcirculation of the fundus [13]. Calcium dobesilate is a vascular protective agent, which can effectively reduce the capillary permeability of patients, refrain platelet aggregation, and reduce blood viscosity, thereby effectively ameliorating blood microcirculation [14]. This study found the number of microvascular tumors in the observation after treatment was less than that of the control, the RI value was lower vs. the control, and the EDV and PSV were greater than those of the control, indicating that the combined treatment may improve the microcirculation of patients with branch retinal vein occlusion, which is mainly linked with the improvement of the physiological function of microvascular wall, and reduction of vascular permeability via calcium dobesilate [15]. Meanwhile, the results of this study clarified VEGF and IGF-1 in the observation were lower than those before treatment, but PEDF was elevated vs. the control, indicating that the combined treatment could restrain retinal neovascularization, which may be the main reason for improving fundus microcirculation. At present, it is believed that lowering the blood glucose is the premise of the treatment of NPDR complicated with cataract. Long-term hyperglycemia will damage the blood-retinal barrier and affect the visual function of patients [16] [17]. This study found the blood glucose and blood lipid in the observation were reduced vs. the control after treatment, indicating that the combined drug can better reduce the body's blood glucose and blood lipid levels. While the specific mechanism is unknown, but the author thinks it may be linked with the improvement of microcirculation.

ICAM-1 is an inflammatory factor that participates in the adhesion of monocytes and lymphocytes to endothelial cells and mediates the process of insulin resistance through inflammation [18]. Relevant studies have pointed out patients with diabetes have elevated ICAM-1 [19] [20]. Endothelial cell activation, elevated intercellular adhesion molecules, and enhanced intercellular adhesion in patients with hyperglycemia may be implicated in abnormal ICAM-1. MCP-1 is a momentous chemokine of monocytes, which can be produced by monocytes and macrophages* in vivo*. It can not only participate in the inflammatory process of the body, but also affect the formation of pathological neovascularization [21]. Studies have shown MCP-1 has chemotactic monocyte and T lymphocyte aggregation, and activates monocytes and macrophages [22] [23]. MIF is a proinflammatory factor that performs as an immunomodulator and participates in various pathophysiological processes of inflammation [24]. Some studies have found plasma MIF in diabetic patients is clearly increased, which can affect inflammation in the body [25]. This study found ICAM-1, MCP-1, and MIF in the observation after treatment had clarified changes, indicating that the combined treatment can reduce the body’s inflammatory response. Relevant studies have pointed out the anti-inflammatory effect of calcium dobesilate may be the mechanism for its efficacy in the treatment of NPDR [26], but further research is needed to confirm it. In this study, no clear difference exhibited in the incidence of adverse reactions between the two. Only 1 case of gastrointestinal reaction took place in the observation, which was apparently relieved after receiving effective intervention, indicating that the combination treatment will not elevate the adverse reactions, that is, the safety of the combination treatment is high.

All in all, calcium dobesilate combined with hypoglycemic drugs has a good clinical effect in the treatment of cataract complicated with NPDR, which can effectively reduce blood glucose and lipids in the body, reduce inflammation, and improve the microcirculation of branch retinal vein occlusion lesions.

## Dodatak

### Acknowledgments

Not applicable.

### Funding

Not applicable.

### Conflict of interest statement

All the authors declare that they have no conflict of interest in this work.
